# Designing Nutrition for Health—Incorporating Dietary By-Products into Poultry Feeds to Create Functional Foods with Insights into Health Benefits, Risks, Bioactive Compounds, Food Component Functionality and Safety Regulations

**DOI:** 10.3390/foods12214001

**Published:** 2023-11-01

**Authors:** Petru Alexandru Vlaicu, Arabela Elena Untea, Iulia Varzaru, Mihaela Saracila, Alexandra Gabriela Oancea

**Affiliations:** Feed and Food Quality Department, National Research and Development Institute for Animal Nutrition and Biology, 077015 Balotesti, Romania; arabela.untea@ibna.ro (A.E.U.); iulia_maros@yahoo.com (I.V.); mihaela.saracila@ibna.ro (M.S.); alexandra.oancea@ibna.ro (A.G.O.)

**Keywords:** antioxidants, bioavailability, food legislation, methods, human health, bioactive compounds, food waste, animal diets

## Abstract

This review delves into the concept of nutrition by design, exploring the relationship between poultry production, the utilization of dietary by-products to create functional foods, and their impact on human health. Functional foods are defined as products that extend beyond their basic nutritional value, offering potential benefits in disease prevention and management. Various methods, including extraction, fermentation, enrichment, biotechnology, and nanotechnology, are employed to obtain bioactive compounds for these functional foods. This review also examines the innovative approach of enhancing livestock diets to create functional foods through animal-based methods. Bioactive compounds found in these functional foods, such as essential fatty acids, antioxidants, carotenoids, minerals, vitamins, and bioactive peptides, are highlighted for their potential in promoting well-being and mitigating chronic diseases. Additionally, the review explores the functionality of food components within these products, emphasizing the critical roles of bioaccessibility, bioactivity, and bioavailability in promoting health. The importance of considering key aspects in the design of enhanced poultry diets for functional food production is thoroughly reviewed. The safety of these foods through the establishment of regulations and guidelines was reviewed. It is concluded that the integration of nutrition by design principles empowers individuals to make informed choices that can prioritize their health and well-being. By incorporating functional foods rich in bioactive compounds, consumers can proactively take steps to prevent and manage health issues, ultimately contributing to a healthier society and lifestyle.

## 1. Introduction

In a world where health and well-being have become paramount concerns, the concept of functional foods has gained significant attention. They offer a promising path towards improving human health and preventing various diseases, all through the power of what we eat [1]. Functional foods represent a bridge between the sustenance we seek from our daily meals and the therapeutic potential embedded within the foods we consume. In simple terms, these are foods intentionally designed or modified to offer additional health benefits beyond basic nutrition [2]. They are fortified with bioactive compounds, such as vitamins, minerals, antioxidants, probiotics, fatty acids, and phytochemicals, which play a pivotal role in promoting health and well-being. Functional foods are a powerful tool that can be used to enhance our health [3], representing the realization of Hippocrates’ ancient adage, “Let food be thy medicine”. They have a profound impact on human health when integrated into a balanced diet, offering a spectrum of health benefits that extend far beyond basic nutrition [4,5], enhancing the immune system, promoting digestive health, mitigating inflammation, enhancing cognitive function, and mitigatemitigating the risk of chronic diseases [5]. For instance, the omega-3 essential fatty acids from animal-derived products are renowned for their ability to reduce the risk of cardiovascular diseases by lowering blood pressure and cholesterol levels, as reported in a clinical study [6]. In addition, the probiotics present in yogurt, which nurture gut health, help digestion, and extend beyond mere taste to functional excellence, are also beneficial [7]. The antioxidants from berries protect brain cells from oxidative stress and may help to stave off age-related cognitive decline [8,9]. To obtain the benefits of functional foods, we need to incorporate them into our daily diets. Fruits, vegetables, wholegrains, berries, leaves, and plants are teeming with antioxidants [10,11,12], while many seeds like chia, flax, and almonds are veritable reservoirs of vitamins and heart-healthy fats [13,14]. In some cases, functional components are deliberately added to foods during processing or manufacturing to enhance their nutritional value. This approach is commonplace with staple foods like cereals, where vitamins and minerals are introduced to combat nutrient deficiencies [15]. In other situations where dietary intake falls short of meeting specific health goals, supplements emerge as a valuable source of functional components. Food waste as an enrichment in bakery food formulation was also successfully achieved [16,17], while the by-products were used to obtain animal-derived products enriched with different essential nutrients [18,19,20].

To reach the full potential of functional foods, various methods and operations are employed to enhance the contents of bioactive compounds, from technological, advanced, and digital to animal nutrition [21,22]. These methods underscore the dynamic nature of the functional food landscape, where scientific ingenuity, sustainable agriculture, and the principles of the circular economy converge to provide a wide range of options for health-conscious consumers in the farm-to-fork journey.

To ensure and validate the potential health implications of functional foods, they have not gone unnoticed by regulatory authorities worldwide. Clinical study testing, scientific validation, transparent labelling, safety assessment, and quality control are required to ensure the safety and efficacy of these products and to safeguard consumers against misleading claims. Although legislative bodies have formulated some regulations, they are not generally applicable, and this aspect might be one reason for consumers’ low interest in functional foods or lack of knowledge about their benefits.

In this context, this review aims to summarize the concept of nutrition by designing functional foods, the methods and operations used to obtain them, the existing definitions of functional foods, health benefits and risks associated with the consumption of functional foods, the bioavailability, bioaccessibility and bioactivity of functional components, and legislative regulations of functional foods.

## 2. Methods and Processing Operations Used to Obtain Functional Foods

Obtaining functional foods involves a sophisticated interplay of methods and processing operations designed to obtain the full potential of natural ingredients. These techniques are integral to enhancing the bioavailability of bioactive compounds, ensuring that functional foods can truly deliver on their promise of promoting human health and well-being [22]. Various methods, including extraction, encapsulation, and fortification, play a pivotal role in the creation of functional foods by concentrating bioactive components and enhancing their absorption within the human body (Table 1). From freeze-drying to fermentation, a diverse array of processing operations is employed to obtain functional foods, and each method is carefully selected to maximize the nutritional benefits and bioaccessibility of these health-promoting products [23]. However, when employing methodologies that enhance a firm’s comprehension of customer motivations and values, the food industry should consider numerous variables when developing or reengineering functional products, including sensory appeal, stability, pricing, chemical composition, functional properties, and convenience, as highlighted by [24]. The process to create a functional food involves an array of methods and processing operations, spanning traditional agricultural practices to cutting-edge technological innovations. According to the literature data in Table 1, the most common methods and operations used to produce functional foods are classified.

Going through all these methods and operations, technological methods are the easiest to use and apply, and, of the functional foods, milk and bakery products are among the most common and easily obtainable functional foods for several reasons. Milk is a staple in many diets worldwide, and bakery products such as bread, cereals, and pastries are also universal [52]. These foods have a long history of consumption, are generally well accepted by consumers, and encompass a wide range of items, from milk and yogurt to various types of bread, cereal, and pastries [53]. This diversity allows for the creation of a broad spectrum of functional options to cater to different consumer preferences and dietary needs. These products are also cheaper, spread worldwide, and hold cultural and dietary significance, further increasing their consumption which encourages the incorporation of functional variations into traditional diets [54].

On the other hand, designing functional foods of animal origin through animal nutrition studies can be considered an advanced method because they offer unique advantages over certain technological methods [55]. Animal nutrition allows for the natural enrichment of animal-derived products through diet manipulation [56]. This means that the functional components are incorporated into the animal’s tissues in a biologically relevant and natural manner. For example, omega-3 fatty acids and antioxidant-rich diets used in poultry farming resulted in chicken or eggs naturally enriched with these health-promoting fats [9,18,57]. Animal nutrition studies also require a deep understanding of animal physiology, dietary requirements, and the interactions between nutrients and bioactive compounds. This knowledge is based on scientific research, precision, and design and is continuously evolving. In contrast, some technological methods focus on isolating specific compounds, potentially missing out on the benefits of natural combinations, which often work synergistically to enhance health benefits [44,45,58,59]. Animal nutrition studies involve precise control over the animal’s diet and nutritional intake to achieve the desired functional properties. This level of control allows for the targeted enrichment of specific bioactive compounds in animal-derived products. Products obtained through animal nutrition often receive positive consumer perception. Consumers are generally more receptive to naturally enriched products compared to those with added or fortified ingredients, which can be seen as less natural [60]. This natural enrichment is often valued by consumers seeking more minimally processed food products.

Animal nutrition studies can also play a role in improving the sustainability of animal farming, and reducing the environmental impact of livestock production, such as minimizing waste pollution or methane emissions, is an area of active research [61]. Further, in many cases, modifying animal diets to obtain functional foods can be a cost-effective approach compared to some technological methods that require specialized equipment and processes [51,62]. Lastly, designing animal nutrition studies also considers the health and welfare of the animals, ensuring that the dietary modifications do not harm the animals’ well-being and align with ethical and animal welfare concerns.

## 3. Nutrition by Design and Functional Foods

The field of nutrition has evolved significantly over the years, shifting its focus from merely meeting basic nutritional requirements to exploring the potential benefits of optimized animal diets and functional foods for human health. As the understanding of the intricate relationship between animal diet and human health continues to expand, researchers and nutritionists have started to embrace a new approach known as nutrition by design [63]. This approach entails the intentional and strategic formulation of enhanced animal diets and functional foods to promote optimal human health and well-being through the utilization of dietary by-products, which are often overlooked resources [64]. Functional foods, fortified with bioactive compounds and specific nutrients recycled from vegetable wastes, provide additional health benefits beyond basic nutrition, targeting specific areas such as immune function, cardiovascular health, and cognitive performance [65]. Animal diets, on the other hand, play a crucial role in enhancing the nutritional value of animal-derived products consumed by humans, such as meat, milk, and eggs. By optimizing animal nutrition through the inclusion of dietary by-products, the nutritional composition of these products can be enriched, obtaining a functional food of animal origin which further contributes to human health [66].

### 3.1. Nutrition by Design

The concept of nutrition by design, in the context of obtaining functional foods, revolves around the deliberate and strategic formulation of food products to deliver specific health benefits beyond their basic nutritional value [67]. It entails a thorough understanding of the interactions between nutrients and bioactive compounds in food and their potential effects on human health. By incorporating these functional components into foods, nutrition by design aims to promote wellness, prevent diseases, and improve overall quality of life [68]. The idea of nutrition by design involves identifying and selecting specific nutrients or bioactive compounds with scientifically proven health-promoting properties that can be added into certain foods to enhance their functional characteristics [63,69]. A crucial aspect of nutrition by design is the integration of scientific research and evidence-based approaches to support health claims associated with functional foods. Rigorous studies have been conducted to understand the mechanisms of action, bioavailability, and bioefficiency of bioactive compounds [70,71]. This scientific foundation provides credibility to the claims made about the functional foods’ health benefits, enabling consumers to make informed choices about their dietary preferences. Nutrition by design involves identifying the most potent sources of these bioactive compounds like antioxidants, polyphenols, omega-3 fatty acids, and other phytochemicals, and optimizing their levels in food products to deliver targeted health benefits [72]. For instance, resveratrol is a polyphenol found in grapes, and it has been associated with various health benefits, including cardiovascular protection and anti-inflammatory properties [73]. Omega-3 fatty acids are another example of bioactive compounds commonly used in functional food design, known for their role in supporting heart health, brain function, and reducing inflammation [74,75]. Apart from individual compounds, nutrition by design also explores the concept of food synergy which refers to the interaction of multiple nutrients and bioactive compounds in a whole food matrix [76], which can create enhanced health effects compared to isolated components [77]. For example, the combination of carotenoids, vitamin C, and fibre in fruits and vegetables exemplifies the power of food synergy in promoting antioxidant activity and overall health [78]. Other examples are given by use of different combinations of by-products, wastes, or co-products, like flaxseed with sea buckthorn or rosehip [14,18], cranberry leaves and walnut meal [9,57], olive mill wastewater and grape pomace [79], and sea buckthorn leaf and chromium [80] as effective sources of essential nutrients and bioactive compounds in poultry products with additional dietary bioactive compounds. In the context of nutrition by design, it is essential to consider food processing techniques because some functional components may be sensitive to heat or other processing methods, affecting their bioavailability and potential health benefits [81].

Nutrition by design also considers the sensory attributes of functional foods like taste, texture, aroma, and appearance, which play vital roles in consumer acceptance and compliance. A food product may be packed with bioactive compounds, but if it lacks palatability, consumers may not incorporate it into their diets regularly [82]. Overall, this concept also recognizes the importance of personalized nutrition catering to individualized health needs based on genetic profiles, population incomes, and dietary preferences. By combining scientific knowledge, innovation, and consumer preferences, nutrition by design plays a vital role in developing functional foods that contribute to improved health and well-being.

### 3.2. Functional Foods

The concept of functional foods originated in Japan in the early 1980s, where the term “Foods for Specified Health Uses” (FOSHU) was introduced. FOSHU products underwent rigorous scientific evaluation to demonstrate their health-promoting properties. This approach paved the way for functional foods to enter the market and sparked interest worldwide [83]. In 1994, the International Food Information Council (IFIC) introduced the term “functional foods” to encompass similar products globally [84]. However, in 2015, Japan introduced a novel food labelling system termed ‘Food with Function Claims (FFC)’. Under this system, industries and agricultural producers have the autonomy to evaluate the scientific evidence regarding their food products and their health-related claims [85]. This empowers consumers to make informed choices. This new approach led to the development of various functional foods with more flexible health claims compared to the FOSHU system, which did not require governmental approval for such claims. While the FOSHU system does not endorse structural–functional health claims, the FFC system permits them. This allows for the rewording of health claims, like changing ‘omega n-3 uptake’ in FOSHU to more appealing phrases such as ‘cardiovascular function’ or ‘heart function’ within the FFC system. Both FOSHU and FFC mandate substantial evidence from clinical studies. However, the FFC system adopts more adaptable protocols and does not necessitate dose-dependent studies.

An analysis of the existing literature on functional foods has highlighted that although the term “functional food” has been defined multiple times, there is currently no universally accepted definition for this category of foods [86]. In most countries, there is no legislative definition of the term, and drawing a clear distinction between conventional and functional foods presents a challenge, even for nutritionists and food experts. Furthermore, European legislation does not consider functional foods as specific food categories but rather as a concept [87].

To date, various international authorities, academic bodies, and industry organizations have proposed definitions for functional foods, as shown in Table 2. While some definitions simply suggest that any food, if marketed with appropriate positioning, can be considered a functional food, others are more complex and argue that only foods fortified, enriched, or improved with a component that provides a health benefit beyond basic nutrition can be classified as functional foods.

In the European Union, functional foods are conceptual rather than designated as a specific food category. The European Commission’s initiatives, such as the Concerted Action on Functional Food Science in Europe (FUFOSE) and the European Food Safety Authority (EFSA), define functional foods as those that beneficially impact body functions beyond nutrition, contributing to improved health or disease risk reduction [118]. In the United States, functional foods do not have a separate regulatory category. The Food and Drug Administration (FDA) does not provide a specific definition or regulatory framework for functional foods. Generally recognized as safe (GRAS) compounds in functional foods do not require pre-market approval by the FDA. However, non-GRAS compounds or substances with specific properties necessitate review [119]. Post-market supervision involves consumers, healthcare practitioners, and the Federal Trade Commission (FTC).

In Latin America, only Brazil has well-established legislation for functional foods and health claims. Although not officially defined, the concept adopted in Brazil is that functional foods are regular foods with health benefits. The National Health Surveillance Agency (ANVISA) in Brazil assesses claims on a case-by-case basis, focusing on scientific validation and clear communication with consumers [120].

All in all, we can define functional foods as food products formulated or modified with the addition of bioactive compounds, such as essential fatty acids, antioxidants, probiotics, prebiotics, phytochemicals, and minerals or other specific nutrients, to provide additional health benefits beyond basic nutrition. Functional foods are designated to enhance physiological functions when consumed as part of a regular diet, which contributes to their targeted health-promoting effects [69]. These foods are intended to optimize health and well-being, complementing a balanced diet and a healthy lifestyle.

Worldwide, the combined global market value of these items, encompassing functional foods and beverages, was approximately USD 258 billion in 2021. This figure is anticipated to ascend to USD 530 billion by the year 2028 [121]. The scientific foundations of functional foods are established through rigorous scientific research. In addition, our understanding of the bioactive compounds and nutrients present in these foods, as well as their physiological effects and potential impacts on human health, comes as a result of extensive *in vitro* animal studies, and human preclinical and clinical research, conducted to investigate the bioavailability, metabolism, and physiological effects of bioactive compounds and the nutrients underlying the health benefits associated with functional foods [122,123,124,125]. This knowledge serves as a basis for the development and formulation of functional foods with optimized nutritional profiles and potential health-promoting effects.

### 3.3. The Synergism between Nutrition by Design and Functional Foods

**Figure 1:**
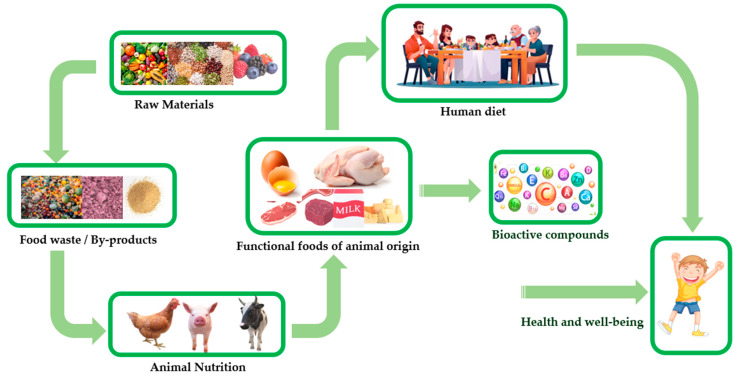
Graphical representation of functional foods obtained through animal nutrition by using food waste and dietary by-products.

Nutrition by design and functional foods are two interrelated concepts that share a common goal of optimizing human health through targeted nutrition [82]. Nutrition by design emphasizes the customization of dietary patterns and nutrient intake to meet specific individual needs, while functional foods focus on incorporating bioactive compounds into the diet to provide additional health benefits beyond basic nutrition [66]. The link between these concepts highlights how they converge to promote personalized and proactive approaches to nutrition. Personalized nutrition based on factors such as age, sex, genetic predisposition, and existing health conditions aims to optimize nutrient intake to support overall health and well-being [126]. This approach considers the dynamic nature of nutritional needs and acknowledges the potential impact of dietary choices on disease prevention. Functional foods also serve as a practical application of nutrition by the design concept by providing a means to incorporate specific bioactive compounds into the diet in a convenient and palatable manner. The development and commercialization of functional foods aligned with nutrition by design principles require a comprehensive understanding of individual nutritional needs and evidence-based research to support health claims [127,128]. Personalized nutrition approaches, such as nutrigenomics, which examine how individual genetic variations influence nutrient metabolism and the response to specific bioactive compounds, play a pivotal role in this integration. Collaboration between food scientists, nutritionists, and healthcare professionals is essential to effectively implement nutrition by design and develop functional foods that align with individualized nutrition recommendations. The joint efforts of these disciplines can ensure that functional foods are not only scientifically validated but also practical, accessible, and appealing to consumers (Figure 1).

## 4. Health Benefits of Bioactive Compounds from Functional Foods in Consumers

As mentioned before, functional foods offer a range of potential health benefits and applications. They provide a diverse array of nutrient-rich options, and hold immense promise in enhancing consumer health and well-being. These foods are recognized for their capacity to not only mitigate the risk of chronic diseases but also to manage existing conditions, such as heart disease, diabetes, and hypertension. Moreover, functional foods, rich in bioactive compounds like omega-3 fatty acids, antioxidants, polyphenols, essential minerals, and vitamins, have been extensively researched for their cognitive-boosting properties, aiding in sharper mental acuity and memory retention. Furthermore, these nutritionally fortified foods play a pivotal role in reinforcing the immune system, bolstering the body’s defence mechanisms against infections and illnesses [129]. Ultimately, the incorporation of functional foods into one’s dietary regimen holds the potential to foster holistic health and vitality, making them an indispensable component of a balanced lifestyle.

### 4.1. Omega-3

Omega-3 fatty acids are a group of essential polyunsaturated fatty acids that play a crucial role in human health. While our bodies cannot synthesize all omega-3 fatty acids, they are essential for various physiological functions and must be obtained through our diet. Fatty fish, flaxseeds, and walnuts are some of the common sources of these valuable nutrients. However, the rise of omega-3 fatty acid-enriched products, including poultry, eggs, and meat, has opened new possibilities for consumers to incorporate these health-promoting compounds into their diets.

Clinical studies have provided substantial evidence supporting the positive effects of eggs and meat enriched in omega-3 fatty acids on various health problems. Numerous randomized controlled trials, observational studies, and systematic reviews have been conducted to investigate the health benefits of consuming omega-3-enriched poultry products, such as eggs and meat [130,131,132]. Some key findings from the scientific literature have revealed that these essential nutrients have a significant impact on various health problems. One of the well-established health benefits of omega-3 fatty acids is their positive impact on cardiovascular health [133]. These essential fatty acids have been shown to reduce blood triglyceride levels, improve LDL cholesterol to HDL cholesterol ratio, provide modest reductions in blood pressure, and improve overall heart function. The regular consumption of omega-3-enriched products, such as eggs and meat from poultry raised on omega-3 fortified diets, exhibit anti-inflammatory properties contributing to cardiovascular health by mitigating inflammation in blood vessels and reducing the risk of atherosclerosis [75]. Clinical research has shown that the increased consumption of omega-3 fatty acids, mostly DHA and EPA, through omega-3-enriched eggs and meat is associated with improved cognitive function and brain health [134]. Studies in both children and adults have found that higher omega-3 intake is linked to better cognitive performance, memory, and attention [135]. Additionally, omega-3s may have a neuroprotective effect, potentially reducing the risk of neurodegenerative diseases like Alzheimer’s [136]. Regarding inflammation and immune support, studies have demonstrated the anti-inflammatory properties of omega-3 fatty acids, which are reflected in the reduced levels of inflammatory markers in the blood of individuals consuming omega-3-enriched products [137]. These findings suggest that omega-3s obtained from eggs and meat can help to modulate the body’s inflammatory response and contribute to improved immune function. Clinical studies have also explored the relationship between omega-3 intake and eye health, particularly in the context of age-related macular degeneration [138]. Observational studies have indicated that higher omega-3 intake, including from eggs and meat, is associated with a decreased risk of age-related macular degeneration progression [139]. While more research is needed to establish causality definitively, these findings support the potential role of omega-3-enriched products in promoting eye health. Moreover, a growing body of research has investigated the link between omega-3 fatty acids and mental health. Several studies, including randomized controlled trials and meta-analyses, suggest that omega-3 supplementation may have a positive impact on mood and may be beneficial in managing symptoms of depression and anxiety [140,141]. While most of this research has focused on fish oil supplements, omega-3-enriched eggs and meat could offer a natural dietary source for these beneficial fatty acids.

### 4.2. Antioxidants

Antioxidants are compounds that play a crucial role in safeguarding our bodies against oxidative stress, a process that can lead to cell damage and contribute to various chronic diseases. These powerful compounds neutralize harmful free radicals, which are unstable molecules that can cause damage to cells and DNA. While antioxidants are naturally found in a wide range of foods, the concept of enriching poultry products, such as eggs and meat, with antioxidants has gained momentum in recent years, especially due to their health benefits through enriched products and their contribution to promoting overall well-being. The primary role of antioxidants is to protect cells from oxidative damage. By neutralizing free radicals, antioxidants help to prevent cellular dysfunction and reduce the risk of oxidative-stress-related diseases [142]. Consuming poultry products enriched with antioxidants can bolster the body’s defence against free radicals, supporting cellular health and overall physiological function [143]. Moreover, oxidative stress can contribute to the development of cardiovascular diseases, such as atherosclerosis and hypertension [144,145].

#### 4.2.1. Hydro-Soluble Antioxidant Compounds

Hydro-soluble antioxidant compounds, particularly polyphenols and vitamin C, play an important role in safeguarding human health and well-being. These compounds are renowned for their remarkable ability to combat oxidative stress, a process characterized by the harmful accumulation of free radicals in the body [146]. By neutralizing these free radicals, polyphenols, and vitamin C help to protect cells from damage, which is closely linked to the prevention of chronic diseases such as heart disease, cancer, and diabetes [146]. Moreover, polyphenols, which are abundant in foods like fruits, vegetables, tea, and red wine, have demonstrated anti-inflammatory properties, aiding a reduction in inflammation-related conditions, and promoting overall immune system function [147].

Incorporating a diet with polyphenol-rich foods and vitamin C is a proactive approach to obtain the health benefits of these hydro-soluble antioxidant compounds. However, enriching animal-derived products with these bioactive compounds is very hard because these products have a lipidic matrix rich in hydro-soluble antioxidant compounds like vitamin C or polyphenols. As was reviewed recently [148], the inclusion of these natural extracts in animals’ feeding diets, although it enhances the oxidative stability of meat and meat products, meeting consumer demand for healthier meat products, the optimum dose of inclusion of polyphenols in animal diets is difficult to define due to the different compositions of phenolic compounds present in by-products. For this reason, this gap must be filled with studies, and after with clinical testing, to monitor the benefits of these obtained foods in humans.

Meanwhile, dietary supplements can also be considered to ensure an adequate intake of these antioxidants, especially for individuals with specific dietary restrictions or limited access to fresh produce. Overall, the role of hydro-soluble antioxidants like polyphenols and vitamin C in promoting human health is indisputable, making them essential components of a balanced and health-conscious lifestyle.

#### 4.2.2. Liposoluble Antioxidant Compounds

Liposoluble antioxidant compounds, like vitamins E and A, are very important in human health and nutrition. These fat-soluble bioactive compounds, derived from both functional foods and the animal kingdom, are veritable guardians of well-being, with the noble effect of protecting our cellular integrity. Vitamin E is an excellent antioxidant which fortifies cell membranes and serves as a sentinel against oxidative damage, which, when procured from dietary sources like nuts, seeds, and verdant foliage, has shown remarkable potential in ameliorating chronic diseases. Likewise, vitamin A has major implications on vision, immunity, and skin radiance, and is mostly obtained from foods such as liver, eggs, and coloured fruits and vegetables. Antioxidants, like vitamin E, have been shown to reduce oxidative damage to blood vessels and cholesterol particles, leading to improved heart health [149]. Regular consumption of poultry eggs and meat enriched with this antioxidant may, therefore, help lower the risk of cardiovascular diseases and support a healthy cardiovascular system [150,151].

Clinical research continues to expose the benefits of these liposoluble antioxidants, revealing their contributions to human health. The scientific evidence has reported that these compounds, when consumed wisely from functional foods or animal origins, offer a tangible promise of enhanced well-being in humans. The consumption of poultry eggs and meat enriched with antioxidants may contribute to healthier and more resilient skin [152]. Antioxidants, especially those found in fruits and vegetables, have been associated with a reduced risk of cognitive impairment and neurodegenerative diseases, such as Alzheimer’s and Parkinson’s [153,154]. By enriching poultry products with antioxidant-rich ingredients, such as certain plant extracts, the potential to support cognitive function and brain health becomes a feasible approach [155,156].

### 4.3. Carotenoids

Carotenoids are a diverse group of compounds, classified into two main categories: carotenes and xanthophylls. Carotenes, such as α-carotene, β-carotene, γ-carotene, and lycopene, are hydrocarbons, and nature boasts approximately 50 different types of carotenes. In contrast, xanthophylls, like β-cryptoxanthin, lutein, zeaxanthin, astaxanthin, fucoxanthin, and peridinin, are carotenoids containing oxygen atoms in various forms [157]. Until 2018, approximately 800 known xanthophylls were identified. Among these, β-carotene, α-carotene, lycopene, β-cryptoxanthin, lutein, and zeaxanthin are the predominant constituents, constituting over 90% of total carotenoids.

Carotenoid distribution within the human body exhibits specific activities. For instance, lutein and zeaxanthin have beneficial effects on the brain and the eye, while lycopene tends to accumulate in the prostate [158]. Lutein and zeaxanthin accumulate in the retina and the lens of the eye, where they act as natural filters against harmful ultraviolet light and protect against oxidative damage. Regular consumption of poultry products enriched with β-carotene, canthaxanthin, astaxanthin, capsanthin, lutein, and zeaxanthin may support vision health and reduce the risk of age-related macular degeneration [159]. The health benefits of carotenoid intake are associated with a reduced prevalence of cardiovascular diseases, diabetes, and cancer, and have previously been attributed mainly to their antioxidant properties and anti-inflammatory effects [160]. Oxidative stress is a significant contributor to skin aging and damage caused by exposure to environmental pollutants and ultraviolet radiation [161]. In 1981, Peto et al. [162] reported a reduction in human cancer rates with dietary β-carotene intake, marking the beginning of several epidemiological studies linking the consumption of green-yellow vegetables and fruits, rich in various carotenoids, to a lower cancer risk, as reported more recently [163]. Clinical trials have demonstrated that natural multi-carotenoid supplements, combined with α-tocopherol, significantly suppressed hepatoma development in a patient with hepatitis virus-induced cirrhosis [164,165]. Moreover, carotenoids have been implicated in the prevention of cardiovascular disease, diabetes, obesity, and various lifestyle-related ailments, while also bolstering the immune system. These findings collectively underscore the manifold advantages of incorporating carotenoid-rich foods into our diets and their far-reaching impacts on human health [166].

### 4.4. Minerals

Macro-minerals, such as calcium, magnesium, phosphorus, sodium, potassium, and sulphur, are formidable players in nerve cell function and blood pressure regulation [167]. On the other hand, micro-minerals like iodine, zinc, selenium, iron, manganese, copper, cobalt, molybdenum, fluoride, chromium, and boron exert their influence on a diverse range of bodily functions, from erythrocyte cell formation to glucose level regulation and immune system fortification [168]. As minerals exert their diverse functionalities within human metabolism and homeostasis, their deficiency can lead to a multitude of prevalent disorders and adverse health symptoms. These minerals must be acquired through dietary sources, yet their presence can vary significantly in different foods and dietary patterns, irrespective of their chemical forms and quantities.

To address mineral deficiencies, the consumption of fortified foods has become more widespread, enhancing the intake of essential minerals in individuals with specific deficiencies, according to the World Health Organization [168]. During the fortification process, it is crucial to assess the bioaccessibility of minerals, considering factors such as dietary inhibitors and enhancers, as well as the methodologies used for determination. Recognizing the importance of minerals, particularly selenium, zinc, and iron, becomes crucial in understanding their pivotal roles in human health prevention [169]. Calcium is primarily obtained from dairy products, with variations ranging from 98 to 1290 mg of calcium per 100 g of cheese [170]. On the contrary, for selenium intake, which is a stalwart antioxidant, standing as a barrier against oxidative stress and fortified immune function, enriched eggs are produced [171]. Zinc the silent hero is predominantly found in meat products (20–60 mg/kg in meat), seafood and fish (>15 mg/kg), and milk (3–5 mg/L) [172]. For this reason, increasing the zinc content in poultry meat can be carried out by manipulating animals’ diets with plants recognized as GRAS by the FDA [12]. Clinical studies have revealed that inadequate zinc intake can compromise the immune response and hinder growth and development [173]. Iron content in meat varies from 2 to 4 mg per 100 g, whereas legumes contain the highest concentration of iron among vegetables, with levels ranging from 7 to 10 mg per 100 g [174]. However, enriched animal products with iron, so far, are not possible as a functional food. This might be due to their potential to become pro-oxidants. In recent years, extensive scientific and technological efforts have been focused on combating mineral malnutrition, which affects both industrialized and developing countries. Iron, zinc, iodine, and selenium deficiencies affect approximately 60%, 30%, 30%, and 15% of the global population, respectively [175]. Furthermore, deficiencies in zinc, iron, iodine, and vitamin A can lead to a distressing 20% mortality rate in children under the age of 5. As we confront these nutritional challenges, potential nutritional strategies must be provided to combat mineral deficiencies and related disorders and diseases. The biofortification of animal-derived products especially poultry, approach might be a promising solution with beneficial impacts on human health.

### 4.5. Vitamin D

Vitamin D is a fat-soluble steroid that plays a crucial role in maintaining overall health and well-being. The two major forms are vitamin D_2_ and vitamin D_3_. Vitamin D_2_ (ergocalciferol) is largely human-made and added to foods, whereas vitamin D_3_ (cholecalciferol) is synthesized in the skin of humans when exposed to sunlight and is often referred to as the “sunshine vitamin”. However, vitamin D deficiency is prevalent, particularly in regions with limited sunlight or during winter months [176]. Hypovitaminosis D is a widespread global health problem associated with levels of 25-hydroxyvitamin D_3_ (25(OH)D_3_) below 20 ng/mL, while the optimal levels in plasma should be in the range of 30–50 ng/mL [177]. It is generally estimated that more than three billion individuals in the world have vitamin D deficiency or insufficiency. To address this issue, enriching foods, including poultry eggs and meat, with vitamin D has gained attention, especially since the COVID-19 era. The health benefits of vitamin D-enriched products found in a few clinical studies have revealed the importance of this essential nutrient [178]. Vitamin D is a key regulator of calcium absorption and utilization in the body as well as phosphorus homeostasis, making it critical for bone health. These clinical studies have reported that adequate vitamin D intake is associated with higher bone mineral density and a reduced risk of fractures, especially in older adults [179,180]; it also has immune-modulating effects, influencing the function of immune cells and reducing the risk of certain infections [179], and reducing the risk of heart disease, hypertension, and other cardiovascular conditions [180,181]. While the evidence is still evolving, ensuring adequate vitamin D levels through enriched products such as eggs and meat from poultry raised on vitamin D-fortified diets can be a convenient way to boost vitamin D intake and support consumers’ health [182]. It must be noted that the response to vitamin D-fortified foods depends on the food matrix, with animal-based foods enriched with vitamin D_3_ exhibiting the most significant impact in maintaining or elevating 25(OH)D_3_ levels [183]. Overall, the effectiveness of enrichment appears to be influenced by factors such as the choice of vitamer for animal feed supplementation (vitamin D2, D3, or 25(OH)D_3_), and the bioaccessibility from the food matrix. Robust human clinical trials are warranted to further explore the role of enriched poultry products in improving vitamin D status [184]. However, European countries have strict legislation about vitamin D supplement used in poultry diets. While the NRC's reported vitamin D requirement of 200 IU/kg of feed, is based on older, slower-growing birds, dating back from 1994, while nowadays the commercial practice far surpasses these levels, ranging between 2,000 and 5,000 IU/kg of feed, aligning with the current EU dietary limit of 5,000 IU/kg. However, this legislation lacks differentiation in the form of vitamin D permitted in diets, with the European Food Safety Authority (EFSA) restricting its addition to water. These aspects make difficult to achieve vitamin D enriched poultry products through animal feeding methods, and this aspect must be changed or clearly stated by regulatory bodies, like the EFSA and the Panel on Additives and Products or Substances used in Animal Feed (FEEDAP).

### 4.6. Bioactive Peptides

Proteins are essential macromolecules that serve a multitude of biological functions in the human body. Recently, there has been growing interest in enzymatic hydrolysis as a method for breaking down proteins into smaller fragments, known as bioactive peptides [185]. These bioactive peptides are known for their health-promoting properties and are increasingly being utilized in the development of functional foods, which can provide various physiological benefits when incorporated into the diet [186]. Literature data reported that diverse protein sources derived from a range of foods have been employed in the production of bioactive peptides. These sources encompass plant-derived proteins, including those from walnut meal, hazelnuts, sesame, perilla seeds, soybeans, common beans, and cauliflower by-products [187,188,189]. Additionally, bioactive peptides have been derived from animal sources like goat, sheep, and bovine milk proteins, eggs, and ham [190,191,192]. Fish and related by-products, such as salmon, stone fish, chub mackerel, turbot skin, shrimp shell discards, and tilapia frame and skin, have also served as sources of these peptides [191,193,194]. Furthermore, microalgae proteins from varieties like blue-green algae, Irish brown seaweed *Ascophyllum nodosum*, and *Tetradesmus obliquus* have been documented as potential sources of bioactive peptides [195,196].

Functional foods of animal origin enriched with bioactive peptides have gained significant attention due to their potential health benefits. Milk-derived and other fermented dairy products are rich sources of bioactive peptides, which can have antioxidant, antihypertensive, and immunomodulatory effects, and opioid-like activities [197]. They contribute to heart health and may help to manage blood pressure. Meat products such as fish (salmon, tuna, and mackerel), beef, chicken, and pork contain bioactive peptides known for their anti-inflammatory properties, contributions to reduce the risk of chronic diseases, arthritis, support of joint health, skin elasticity, and gut function [187]. The biological properties of egg peptides, including but not limited to antioxidant, antimicrobial, antihypertensive, and anticancer effects, are also of great interest. Enzymatic hydrolysis has been established as a promising method for cleaving polypeptide sequences, yielding bioactive peptides that offer both nutritional and therapeutic advantages for human health [198]. While the traditional approaches are commonly used to study the in vitro bioactivity of antioxidant peptides from specific sources, enzymatic hydrolysis and fermentation are favoured over chemical methods due to their safety (GRAS). However, it was recently reviewed by Tadesse et al. [199] that these biochemical methods are not practical for large-scale, cost-effective production, while innovative processing technologies like high hydrostatic pressure, microwave processing, and pulsed electric fields are preferred to yield antioxidant peptides more efficiently, with improved bioactivity, shorter production times, and lower costs compared to biochemical methods.

Regulatory guidelines vary, necessitating adherence and clinical trials to substantiate health claims. Ongoing research focuses on optimizing processes, identifying new peptide sources, and unravelling the mechanisms behind their benefits, addressing challenges such as standardization, taste, and consumer acceptance. In the paper of Daliri et al. [184], the beneficial effects of bioactive peptides were largely discussed. However, the authors stated that factors such as the possibility of allergenicity, cytotoxicity, and the stability of the peptides during gastrointestinal digestion still need to be researched.

## 5. Potential Risks and Drawbacks Associated with the Consumption of Functional Foods Enriched with Bioactive Compounds

Functional foods have garnered significant popularity as consumers seek ways to improve their health through dietary choices. These foods often contain bioactive compounds designed to offer specific health benefits and promote well-being [200]. While the benefits have been reviewed for specific health conditions, it is essential to address the relatively scarce comprehensive in vivo research that confirms these health claims. The current body of literature relies heavily on in vitro studies, particularly those examining single food constituents such as micronutrients, and presumes their safety due to their derivation from commonly used food sources [201,202]. However, it is crucial to acknowledge the potential health risks associated with these ingredients and provide comprehensive information on possible unwanted side effects, especially on product labels. Allergic reactions to certain bioactive compounds, like those from nuts, soy, or fruits, can pose a serious concern. Ensuring the functionality and safety of food products requires validation from clinical studies rather than solely in vitro investigations [200]. Claims about the nutritional and health benefits of foods also play a significant role in guiding consumers toward maintaining a healthy diet [203].

The risks and adverse reactions to functional foods can be categorized similarly to those associated with conventional foods, encompassing both toxic and non-toxic responses. Toxic reactions, such as carcinogenicity, may occur in products containing carcinogenic substances or high doses of added ingredients. Unfavourable reactions can result from overconsumption, especially of antioxidants, resulting in nutrient imbalances or toxicity, potentially leading to adverse effects [204]. Surprisingly, even functional food components with recognized health benefits, like vitamin A or vitamin-carotene, may induce adverse effects when consumed in high doses [205].

The effects of functional foods can be influenced by the concentration of each component and the potential synergistic or antagonistic effects of various molecules. For example, high levels of genistein, a soy phytoestrogen, have been linked to the promotion of certain tumour types in animals, contrary to the expected health benefits [206,207]. It is important to consider the potential toxicity of various polyphenols and their impact on cell lines, like concentrations of catechin of over 150 μmol/L [208]. Moreover, bioactive compounds may interact with medications or other supplements, leading to unintended consequences. For instance, some bioactive compounds can interfere with the absorption of medications or enhance their effects, potentially causing complications for individuals on specific drug regimens. Food hypersensitivity, which encompasses food allergy and food intolerance, can lead to various adverse reactions. Food allergy is an immune system reaction that triggers the release of antibodies against specific allergenic proteins in foods [209]. Chemical intolerances are related to reactions to food additives, including artificial colours, preservatives, and other substances. Another risk associated with functional foods that should be considered is the potential for misleading marketing and exaggerated health claims, as found by Diaz et al. [210]. In the absence of rigorous scientific evaluation, manufacturers may make unverified claims about the health benefits of their products, leading consumers to believe they can address specific health issues or replace traditional medical treatments. This can create false expectations and undermine the role of healthcare providers in managing health conditions.

The need for rigorous scientific evaluation is paramount in addressing these risks and ensuring the safety and efficacy of functional foods enriched with bioactive compounds. Clinical trials and studies are essential to provide concrete evidence regarding the effects of these foods on human health. These studies should encompass a wide range of participants, including diverse populations and age groups, to account for variations in how different individuals may respond to these products. Furthermore, regulatory bodies should establish and enforce stringent standards for the labelling and marketing of functional foods. This includes requiring clear and accurate information about the bioactive compounds in the product, their recommended intake levels, and potential side effects. Ensuring that health claims are substantiated by robust scientific evidence can protect consumers from misleading information.

## 6. Bioaccessibility, Bioactivity, and Bioavailability as Food Component Functionalities

The efficacy of food bioactive compounds hinges on various factors such as metabolomics, nutrigenomics, bioavailability, and their retention within the food matrix [211]. As mentioned above, functional foods are produced by incorporating compounds into traditional or innovative food products that alter their properties, such as structure or binding, and confer health benefits. The preparation of such foods necessitates the consideration of various factors, including the selection of appropriate sources added, identification of target compounds, choice of recovery methods and technologies, and, when required, toxicological assessments. Furthermore, comprehensive evaluations involving bioaccessibility, bioactivity, and stability assessments are crucial [212]. However, sometimes the terms bioaccessibility, bioactivity, and bioavailability are used interchangeably to describe similar and pertinent functions, although they have different definitions.

Bioavailability encompasses various meanings depending on the field of study. In pharmacology, it pertains to the absorption rate and extent of therapeutic molecules [213]. In the food domain, it relates to the proportion of ingested food compounds reaching the systemic circulation [214]. Various factors, including the food matrix, ingredients, and digestive processes, influence bioavailability. Tailoring diets to different life stages ensures optimal nutrient balance. Bioavailability is vital for defining functional foods and claims related to health or nutrition. It depends on factors like consumer characteristics, nutrient form, and food matrix. Larger molecules, like fats and proteins, have high bioavailability, while variability can result from food matrix differences, processing, or enzyme activity [70].

Bioaccessibility, a component of bioavailability, is a critical concept in nutrition and it refers to a compound’s release from the food matrix in the gastrointestinal tract, ready for absorption [215]. Before becoming bioavailable, food components need to be initially released from the food matrix and undergo digestion. The first step is ingestion, when we eat food, followed by the process of digestion which starts in the mouth, where mechanical and chemical processes begin breaking down the food into smaller particles. The second step is stomach digestion, where food is mixed with gastric juices, which contain hydrochloric acid and digestive enzymes. These substances help to break down the food further and initiate the release of bioactive molecules from the food matrix. The partially digested food then moves into the small intestine, where the pancreas releases digestive enzymes, and the gallbladder releases bile [216]. These enzymes and bile work together to further break down food, releasing bioactive compounds in the process. Lastly, the released bioactive molecules, in an absorbed form, move across the intestinal lining and into the bloodstream [114,217]. This is where these molecules become bioavailable and can exert their physiological effects in the body. Factors like compound interactions also affect bioaccessibility. Recently, it was mentioned [218] that before making health claims about food compounds, the impact of digestion on the stability and function of bioactive compounds must be assessed because bioaccessibility assessment typically involves simulating small intestinal digestion through in vitro methods. However, it is reported that standard experimental models sometimes fail to distinguish between bioaccessibility and bioavailability efficiency [219], making it challenging for bioaccessibility to become a crucial parameter for functional foods.

Bioactivity (in vivo, ex vivo, and in vitro) occurs after nutrient absorption and involves various physiological responses, such as anti-inflammatory or antioxidant effects [214,220]. Digestibility concerns focus on food components transformed into accessible forms during digestion. The scientific basis for food health claims often relies on bioactivity. Some authors reported that different methods, including in vitro, in vivo, and ex vivo, are used to evaluate the bioactivity of food products [214,221]. However, ethical and practical challenges arise when determining bioactivity at specific organ sites, which makes bioavailability assessment necessary through in vivo trials [170].

**Figure 2:**
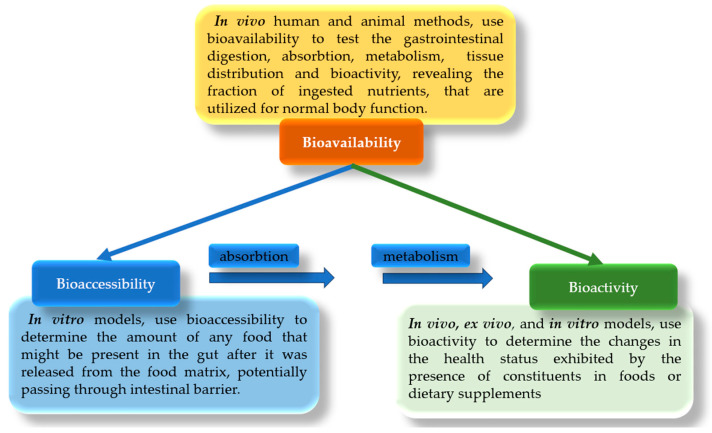
General representation of the differences between bioavailability, bioaccessibility, and bioactivity of food components, using in vivo, ex vivo, and in vitro models.

Creating fortified foods with functional components involves a comprehensive evaluation that encompasses several key elements. This process entails the careful selection of a suitable source, the identification of bioactive compounds, the application of separation and recovery methods, the conduction of toxicological assessments, and the subsequent measurements of stability, activity, and bioaccessibility. For this, it is crucial to establish precise definitions for the terms bioavailability, bioaccessibility, and bioactivity (Figure 2), as these terms are frequently used interchangeably to describe similar functions.

## 7. Major By-Products Used to Obtain Poultry-Derived Functional Foods and Their Main Bioactive Compounds and Essential Nutrients

The bioactive compounds and essential nutrients found in by-products vary depending on the specific by-product obtained. In Table 3, the major by-products used, and their major bioactive compounds are presented among the most commonly studied by-products to produce functional foods in animal nutrition studies.

These by-products have been proven to enhance the contents of beneficial components such as fatty acids, antioxidants, polyphenols, and extend the shelf-life of poultry products, contributing to improved nutritional value and overall quality, resulting in a designed functional food product. The use of diverse by-products in animal diets contributes to sustainable agriculture by reducing food waste and promoting circular economies. However, it is essential to note that the effectiveness of these by-products in designing functional products may vary depending on factors such as the specific by-products used, their inclusion levels in the diet, and the targeted animal species [253].

## 8. Enhancing Poultry Diets with Dietary By-Products and Aspects That Should Be Considered to Obtain Functional Foods

Enhanced animal diets with dietary by-product ingredients are gaining popularity as a sustainable and cost-effective approach in animal nutrition. Dietary by-products are residual materials from food processing industries, which were previously considered waste but are now recognized for their nutritional value and potential benefits. Incorporating dietary by-products into animal diets helps to reduce food waste, promoting eco-friendly practices [254]. Approximately 1.3 billion tons of waste are generated annually worldwide due to agro-industrial processes and losses across the entire production chain, spanning fields, processing, logistics, retail, and consumption [255]. This presents a concerning scenario, particularly in a world where hunger remains a pressing issue, impacting over 1.2 billion individuals. These individuals not only lack access to sufficient food but also face uncertainty regarding the safety, nutritional adequacy, and reliability of their diets. The emergence of the COVID-19 pandemic has further intensified this predicament, resulting in a higher number of individuals experiencing food insecurity [256]. As a result, there is an amplified need to implement more effective strategies to address both waste and hunger challenges. Common dietary (functional ingredients) by-products include fruit and vegetable peels, meals and cakes resulting after oil extraction, brewers’ grains, and spent grains from the brewing industry. By-product ingredients can vary depending on the specific food processing industry and the availability of resources in a particular region [250]. The utilization of dietary by-products in animal diets offers economic advantages, as they are typically more affordable than traditional feed ingredients. This can significantly reduce production costs for farmers and livestock producers, contributing to sustainable agriculture practices. However, proper evaluation and processing are necessary to ensure that dietary by-products are safe and beneficial for animal consumption since some by-products may contain anti-nutritional factors if not adequately processed or balanced in the diet [257].

In the context of designing animal diets and functional food, this research centres on a holistic understanding of animal physiology and dietary requirements to optimize their health, performance, and product quality. It involves a comprehensive approach that considers the specific nutritional needs of each animal species, their life stages, and their evolutionary adaptations, ultimately leading to the formulation of tailored diets and functional foods that offer numerous benefits to the end-consumers [258]. This knowledge is crucial in understanding how animals naturally process and utilize nutrients. Metabolic processes play a significant role in determining the nutritional requirements of animals. Efficiently formulated and balanced diets contribute to the overall health and well-being of animals, reducing the need for antibiotics and veterinary interventions, and promoting sustainable agricultural practices, where animal welfare and environmental concerns are carefully balanced [259]. Enhanced animal diets play a critical role in producing functional foods that cater to specific dietary requirements or health conditions. Scientific research and the understanding of animal physiology allow for the identification of bioactive compounds and functional ingredients that can be incorporated into animal diets and ultimately transferred to the consumer when consumed as animal-derived products. However, when enhancing animal diets with dietary by-products, some key aspects should be considered.

### 8.1. Nutrient Composition of Diets

This aspect is essential for meeting animals’ nutritional needs and optimizing health. It involves selecting specific nutrients, bioactive compounds, and functional ingredients tailored to each species’ requirements, considering factors like age and health. Proteins, carbohydrates, fats, vitamins, minerals, and water are crucial components for growth and development while bioactive compounds like antioxidants and probiotics offer health benefits. Regulatory bodies such as the Association of American Feed Control Officials (AAFCO) and the European Feed Manufacturers’ Federation (FEFAC) ensure that diets and feed ingredients meet nutritional standards, ensuring animal health and safety [260].

### 8.2. Nutritional Adequacy of Diets

Nutritional adequacy is a critical aspect of designing diets or dietary interventions that meet the recommended levels of essential nutrients, vitamins, and minerals necessary for supporting optimal physiological functions. By meeting the recommended nutrient intake levels, diets can effectively support animals’ physiological processes, promoting growth, development, and overall performance [260]. Nutritional adequacy plays a key role in preventing nutrient deficiencies or excesses, both of which can lead to various animal health issues. Balanced diets support growth, development, and performance, preventing deficiencies or excesses that may harm an animal’s health [261].

### 8.3. Bioavailability and Absorption

As we mentioned above, bioavailability refers to the proportion of nutrients that are readily absorbed and utilized by the body after consumption, while absorption pertains to the process by which nutrients are taken up by the body’s cells [213]. When formulating diets or assessing nutritional adequacy, it is essential to consider how efficiently nutrients from different feed sources can be absorbed and utilized by the body. Factors such as the chemical form of nutrients, interactions with other compounds present in the diet, and an individual’s physiological status can influence the bioavailability and absorption of nutrients. Some nutrients may have higher bioavailability when obtained from certain feed sources or when consumed in specific forms [262]. As reported by Ammerman et al. [262], understanding the bioavailability and absorption of nutrients helps to optimize diet planning, reducing the risk of deficiencies and promoting overall health.

### 8.4. Individual Variability in Animal Nutrition

In the animal nutrition field, individual variability plays a significant role in designing effective nutrition interventions. Acknowledging the diverse nutrient requirements, metabolism, genetics, and health status of each animal is essential for promoting optimal health and performance [263]. Personalized nutrition approaches, like precision nutrition, nutrigenomics, and nutrigenetics, offer valuable tools to customize dietary recommendations based on an animal’s distinct genetic profile and health attributes [264]. Animals within a species can exhibit variations in their nutritional needs due to factors such as age, weight, activity level, and overall health. By adjusting nutrient composition and levels, nutritionists can optimize nutrient absorption and utilization, ensuring better overall health and performance. As reviewed previously by de Toro-Martin et al. [264], implementing personalized nutrition not only enhances individual animal health but also leads to improved production outcomes in agricultural settings and better overall animal welfare in various husbandry systems.

### 8.5. Evidence-Based Practice

Evidence-based practice uses scientific research to guide dietary interventions. Trials and studies provide reliable data, ensuring that dietary recommendations are based on solid evidence rather than anecdotal information. Literature data provide evidence for the effectiveness of various by-products as valuable additions to poultry diets. The incorporation of these by-products into animal feed formulations can result in several positive outcomes being recognized as valuable sources of protein and essential nutrients, offering a sustainable and cost-effective solution for enhancing production performance in the agricultural industry. The use of dietary by-products, as rich sources of essential nutrients, showed positive effects on growth and weight gain in poultry, and led to the development of functional foods like eggs and meat.

Functional foods derived from poultry have been created by modifying the diets of these birds using bioactive compounds or by-products. These dietary manipulations result in poultry products with enhanced nutritional profiles and potential health benefits. For example, poultry fed diets rich in omega-3 fatty acids from various sources produce meat and eggs with elevated levels of these heart-healthy fatty acids, which are known to benefit cardiovascular health, brain function, and reduce inflammation [265]. When poultry, such as laying hens or broiler chickens, are fed flaxseed meal, their egg yolks accumulate significant amounts of alpha-linolenic acid (ALA), eicosapentaenoic acid (EPA), and docosahexaenoic acid (DHA) when flaxseed meal constitutes up to 20% of their diet [14,266,267]. This dietary modification also improves the overall quality of the products and can reduce cholesterol levels in some cases. Eggs and poultry meat are suitable candidates for fatty acid profile modification to increase the content of n-3 polyunsaturated fatty acids (PUFA) like ALA, EPA, and DHA, which are essential for a balanced diet. Regulations in the European Union require foods to contain at least 0.3 g ALA per 100 g and/or at least 40 mg EPA + DHA per 100 g to be labelled as a source of omega-3 fatty acids, and at least 0.6 g ALA per 100 g and/or at least 80 mg EPA + DHA per 100 g to be considered a rich source of omega-3 fatty acids [268]. Poultry, unlike humans, can efficiently convert ALA into DHA, making them an excellent source of this essential fatty acid when fed flaxseed or other ALA-rich by-products. However, care must be taken when incorporating over 10% flaxseed meal into poultry diets, as it may negatively impact production performance and product shelf-life [269]. Other by-products like rapeseed meal, cottonseed meal, camelina meal/cake, and rosehip meal have also been effective in enhancing the nutritional content of poultry products when added to their diets [229].

Incorporating antioxidant-rich by-products derived from fruits, extracts, and plants into poultry diets can lead to meat and eggs with higher levels of health-promoting compounds. These compounds help to neutralize harmful free radicals, reduce oxidative stress, and lower the risk of chronic diseases [9,12,240]. Additionally, supplementing laying hens’ diets with natural sources of carotenoids like lutein and zeaxanthin can result in eggs with increased levels of these compounds, benefiting eye health and potentially preventing age-related macular degeneration [148,270,271]. Introducing selenium-enriched feed to poultry can turn poultry products into reliable sources of this essential trace mineral. Selenium plays a crucial role in antioxidant defence and immune function, contributing to overall health [272,273]. Poultry products can also be fortified with vitamin D by including it in their diets, promoting bone health, immune function, and various metabolic processes [274]. Furthermore, incorporating live microorganisms into poultry diets can enhance gut health and potentially improve the nutritional value of meat and eggs. This can lead to better digestion and immune function in both the birds and consumers [275], especially due to low or no antibiotic usage.

In essence, functional foods from poultry represent a convergence of scientific innovation and nutritional understanding, offering a tangible way to improve human health through designing diets. As research continues to explore the potential of these foods, their range of benefits is likely to expand, revolutionizing how we perceive and approach human and animal nutrition.

## 9. Safety and Regulation of Functional Foods

Ensuring the safety and efficacy of functional foods is of utmost importance. The safety and regulation of functional foods are of high importance to ensure that these products are safe for consumption and deliver the claimed health benefits without causing harm to consumers. In Europe, regulatory bodies, such as the European Food Safety Authority [276], have established few guidelines and regulations for functional foods. These guidelines specify the permissible levels of bioactive compounds, nutrients, and health claims that can be made on product labels ((Regulation (EC) No 1924/2006; U.S. Food and Drug Administration) [277].

Functional foods, like any other food product, must undergo extensive testing and research, and rigorous safety and efficacy assessments before they can be marketed and sold to consumers. This involves toxicity studies, clinical trials, and post-market surveillance to monitor any potential adverse effects or interactions with medications, evaluating potential hazards, allergens, contaminants, and other safety concerns associated with the ingredients used in the product.

In the United States, the FDA (Food and Drug Administration) designates certain food ingredients as generally recognized as safe (GRAS), which means they are considered safe for consumption based on a history of safe use or scientific evidence. Manufacturers may self-affirm GRAS status for their ingredients, or they can submit a GRAS notification to the FDA for review.

In the European context, the fundamental principles and regulations governing food law, along with the creation of the European Food Safety Authority (EFSA) and the foundation of food safety standards, are outlined in the EU General Food Law (Regulation (EC) No. 178/2002) [278]. This regulation also encompasses the essential “precautionary principle”, which forms the core of decision-making in matters related to food when uncertainties exist. In some cases, functional foods that contain new ingredients or novel bioactive compounds may require pre-market approval from regulatory agencies before they can be sold to consumers. This approval process typically involves providing scientific evidence of safety and efficacy. Since the EU framework has no concrete definitions to attest functional food status, there are some regulations that define groups of aliments, as presented in Table 4.

## 10. Challenges and Future Directions

While functional foods hold great promise for improving public health, several challenges remain.

Further research is needed to fully understand the mechanisms of action and optimal dosages of bioactive compounds in functional foods. Robust clinical trials and long-term studies are essential for establishing causality and assessing the true health benefits.

In the global marketplace, there are efforts needed to harmonize regulations related to functional foods to facilitate trade while ensuring consumer safety. Organizations like the Codex Alimentarius Commission should also play a role in setting international standards for functional food regulations, safety, and quality. Harmonizing regulations and standards across countries will facilitate the development, marketing, and trade of functional foods, ensuring consumer safety and promoting international collaboration in this field.

Increasing consumer awareness and providing accurate information about functional foods are crucial. Misleading claims and labelling practices can undermine the credibility and potential benefits of functional foods. Functional foods must have accurate and informative labelling to provide consumers with clear information about the product’s ingredients, health claims, and proper usage.

Manufacturers of functional foods must adhere to strict quality control standards and quality control and good manufacturing practices (GMP) to ensure the consistency, purity, and safety of their products. Regular inspections may be conducted to ensure compliance.

After a functional food is approved and introduced to the market, post-market surveillance should be conducted to monitor its safety and efficacy in real-world conditions. This will help to identify any potential long-term or rare adverse effects.

Striking a balance between promoting better health and protecting individuals from potential harm, scientific evaluation, transparent labelling, and responsible marketing is indispensable.

## Figures and Tables

**Table 1:** Methods and processing operations used to obtain functional foods.

Methods/Processing Operations	What Involves	Products	References
Technological methods/operations			
Fortification or enrichment	This method involves adding specific nutrients or bioactive compounds with low nutritional values to a food product, during osmotic dehydration, hydration, and cooking or using pretreatment. However, their bioavailability, stability, or nutrient location in food are scarcely analysed.	cereals, dairy products, flour, condiments	[25]
Fermentation	Fermentation is a natural safe technique for food preservation that enhances the bioavailability and digestibility of nutrients in foods, which are rich in probiotics and beneficial microorganisms. The most common fermentations are lactic acid homofermentation and heterofermentation, butyric acid, mixed acid, propionic acid, and acetic acid.	yogurt, kefir, and sauerkraut; beans, legumes, flours, and some cereals	[26]
Sprouting	Sprouting seeds, grains, or legumes increases their nutrient content, including vitamins, minerals, and antioxidants. This method also increases the digestibility and sensory qualities of sprouts and decreases the levels of anti-nutritional components.	vegetable, fruits, cereals, spices	[27,28]
Extraction	Extracting bioactive compounds from natural sources, like herbs, fruits, or vegetables, to create supplements or functional ingredients for food products.	supplements	[29,30]
Biotechnology	This method allows for the modification of crops to enhance their functional properties, designed to have higher nutrient content or resistance to pests.	genetically modified crops	[31]
Advanced methods/operations			
Food processing techniques	Certain food operations, such as freeze-drying or freeze concentration, can preserve the bioactive compounds in foods while removing water, reduction/elimination, or other unwanted compounds, extending shelf-life, and concentrating nutrients.	juices, fruits, jams	[32,33]
Nanotechnology	Nanotechnology is employed to encapsulate and deliver bioactive compounds like vitamins, minerals, or antioxidants in nano-sized particles. This enhances their stability, solubility, and bioavailability, allowing for controlled release in the body.	bakery, pasta, cereal based products	[34,35]
Microencapsulation	This technique involves coating bioactive compounds with a protective layer, typically a food-grade polymer, to prevent degradation during storage and to increase their stability. Microencapsulated compounds can be added to various food products to make them functional.	phytosterols, fatty acids, polyphenols, other bioactives.	[36,37]
Extrusion	Extrusion is a high-temperature, high-pressure process used to modify the structure of foods. It can increase the availability of bioactive compounds, such as by improving the digestibility of proteins or breaking down complex carbohydrates.	cereal based products, flours,	[38]
Spray drying	Spray drying is used to convert liquid bioactive ingredients into powder form while maintaining their functional properties. This method is commonly employed in the production of powdered supplements and fortified foods.	oils, flours, bioactive compounds.	[39]
High-Pressure processing	Is a non-thermal food processing technique that preserves the nutritional and sensory qualities of foods; reduction/elimination of mycotoxins from foods; improvement of emulsifying properties, gelling capacity and formation, and apparent digestibility. It can be used to maintain the bioactive compounds in fresh foods.	juices, tomatoes, carrots and broccoli, fish, meat, egg, cheese	[40,41]
Pulsed electric field processing	This method uses short bursts of electricity to increase the permeability of cell membranes in foods. This method can enhance the extraction of bioactive compounds from plant materials.	eggs, corn, beetroots, bioactive compounds.	[42,43]
Cold plasma technology	Cold plasma treatment can improve the microbial safety and shelf-life of foods while preserving their nutritional content. It is used in the development of functional foods like fruit juices.	juices, nuts, cereals, fruits, dairy products	[44,45]
Membrane filtration	This method uses selective membranes to separate and concentrate bioactive compounds from food sources, such as proteins, peptides, and polyphenols, for use in functional food products.	fruit and vegetable juices	[46]
Ultrasonication	Ultrasonic waves are employed to break down cell walls and release bioactive compounds from plant materials. This method can be used in the extraction of phytochemicals from fruits and vegetables.	grape pomace, cereals	[47]
Ohmic heating	Ohmic heating uses electrical resistance to heat food products uniformly. It is also used to improve the extraction and retention of bioactive compounds in various foods.	seafood and surimi	[48]
Enzymatic hydrolysis	Is a processing operation which involves the use of specific enzymes to break down complex compounds, such as proteins, into smaller, bioactive peptides. This process enhances the bioavailability of nutrients, making them more readily absorbed by the body.	protein hydrolysates, fish peptides, fruit enzyme extracts	[49,50]
Digital methods/operations			
3D printing	These innovative methods involve customized food design, digitalized and personalized nutrition, the efficient use of raw material, and expansion of the food material source. This technology was developed to design and manufacture food with defined properties and characteristics, which can be employed in the development of functional foods to fulfil consumer demands for more healthy food.	cereals, pasta, meat, fish products, fruits.	[21,22]
Nutritional methods			
Animal nutrition studies	This method focuses on the biology and chemistry of different nutrients, minerals, bioactive compounds, and feed additives related to animal health and the production of animal products with enhanced qualities.	poultry, dairy, pork products	[14,20,51]

**Table 2:** Functional food definitions according to literature data.

Definition	Reference
Foods expected to provide certain health benefits and authorized to carry a label asserting that individuals using them for specific health purposes can anticipate health improvements through their consumption.	[86]
Food containing potentially beneficial products, including any modified food or food ingredient that may offer a health benefit beyond traditional nutrients it contains.	[88]
Food products and beverages derived from natural substances consumed as part of the daily diet and possessing exceptional physiological benefits when ingested.	[89]
Foods that can offer health benefits beyond basic nutrition.	[90]
Foods or food products marketed with a health benefit message.	[91]
Foods derived from natural substances that can be consumed as part of the daily diet and serve to regulate or affect a specific bodily process when ingested.	[92]
Foods similar in appearance to conventional ones, consumed as part of the regular diet, demonstrating physiological benefits and/or reducing the risk of chronic diseases beyond basic nutritional functions.A functional food is either a conventional food or a food that appears similar to a conventional food and is part of a regular diet, providing health benefits and/or reducing the risk of specific chronic diseases beyond its basic nutritional functions.	[93]
Modified foods or food ingredients offering health benefits beyond their traditional nutrients.	[94]
Foods with added ingredients claiming to provide a health benefit to consumers, beyond the benefits offered by regular foods themselves.	[95]
A food product can be considered functional only if, in addition to its basic nutritional impact, it has beneficial effects on one or more functions of the human body, either by improving overall physical conditions and functions or by reducing the risk of disease progression. The quantity consumed and the form of the functional food should be as expected for normal dietary purposes. Therefore, it should not be in the form of a pill or capsule, but rather like regular food.	[96]
Functional foods are formulated products with natural chemicals (or combinations of chemicals) found in many fruits, vegetables, grains, herbs, and spices to provide a health benefit, reduce the risk of certain diseases, or affect a specific organism or process. They go beyond correcting diseases like pellagra and scurvy caused by nutritional deficiencies. Functional foods are similar to novel macro-ingredients, as their formulation is intended to provide a health benefit to consumers. However, functional foods are designed to reduce the risk of specific diseases, such as lung cancer, by eliminating certain ingredients, adding or combining ingredients not typically found in a food product, or by concentrating substances in higher amounts than usual.	[97]
Foods that, due to their physiologically active components, offer benefits beyond basic nutrition and can prevent diseases or promote health.	[98]
Foods to which ingredients with additional health value have been added, and this is announced to consumers.	[99]
A food is functional if a health claim can be made.	[100]
Foods or food components that can have health benefits and reduce the risk of specific diseases or other health problems.	[101]
A food that looks like a conventional food but has physiological benefits and/or reduces the risk of chronic diseases beyond basic nutritional functions.	[102]
A food that is a food and not a medicine, and that is part of a normal diet, providing benefits beyond basic nutrition.	[103]
(1) A natural food to which a component has been added and from which another component has been removed; the nature of one or more components has been modified, as has the bioavailability of one or more components.(2) Foods derived from natural substances that can and should be consumed as part of the daily diet and serve to regulate or affect a specific bodily process when ingested.	[86]
Any food for which a health claim can be made is a functional food.	[104]
Foods that can be part of our daily diet but have properties that offer an additional health benefit.	[105]
Functional foods primarily aid in nutrient provision but additionally offer a special health advantage.	[106]
Foods similar in appearance to conventional foods intended to be consumed as part of a normal diet but have been modified to have physiological roles beyond providing basic nutritional requirements.	[107]
Foods similar in appearance to conventional foods consumed daily in the diet but which, in addition to their basic nutritional value, contain additives or specific properties obtained through processing or other methods for which a physiological/health benefit beyond basic nutrition is claimed.	[108]
A whole food (as opposed to pills, powders, or supplements) that is fortified, enriched, or enhanced with a component having a health benefit beyond basic nutrition.	[100]
Foods that include potentially healthful products, including any modified food or food ingredient that may offer a health benefit beyond the traditional nutrients it contains.	[109]
Foods that can be regularly consumed as part of a normal diet, specially designed to provide a physiological or medical benefit by regulating bodily functions to protect against or delay the onset of diseases such as coronary heart disease, cancer, hypertension, diabetes, and osteoporosis.	[110]
Foods that, in addition to providing known nutrients, can offer other health benefits.	[111]
Any food or food ingredient that may offer a health benefit beyond the traditional nutrients it contains.	[112]
A food with added technologically developed ingredients with a specific health benefit.	[113]
(1) A functional food is or appears similar to a conventional food. It is part of a standard diet and is consumed regularly in normal quantities. It has proven health benefits, reduces the risk of specific chronic diseases or beneficially affects target functions, beyond its basic nutritional functions.(2) A food can be considered functional if it is satisfactorily demonstrated to beneficially affect one or more target functions in the body, beyond adequate nutritional effects, in a manner relevant either to improve health and well-being and/or reduce the risk of illness.	[114]
Food that has a demonstrated benefit for one or more functions of the human body, improving health or well-being, or reducing the risk of illness.	[115]
Food products enriched with special constituents that have advantageous physiological effects.	[116]
Whole foods and fortified, enriched, or enhanced foods with the potential to benefit health when consumed as part of a varied diet, regularly, at effective levels.	[117]
Foods that, with their specific health effects, could indicate a new way of thinking about the relationship between food and health in everyday life.	[118]

**Table 3:** Main bioactive compounds and essential nutrients found in by-product ingredients.

By-Product	Main Bioactive Compounds and Effects	References
Oilseed by-products		
Olive pomace	The residual material left after olive oil extraction is rich in bioactive compounds such as polyphenols, tocopherols, tocotrienols, phytosterols, squalene, triterpenic acids, and unsaturated fatty acids, which collectively offer potential health benefits, including antioxidant, anti-inflammatory, cardioprotective, and immune-supportive effects.	[222]
Sunflower meal	Residue from sunflower oil extraction, a good source of protein, contains a diverse array of main bioactive compounds, including phenolic compounds, phytosterols, tocopherols, and lignans, which contribute to its nutritional and health-promoting properties.	[223]
Canola meal	Canola meal is rich in essential nutrients and bioactive compounds, including protein, fibre, omega-3 and omega-6 fatty acids, and glucosinolates, making it a valuable and health-promoting feed ingredient for animals.	[224]
Peanut shells	Peanut shells are rich in a variety of bioactive compounds and essential nutrients, including polyphenols, flavonoids, dietary fibre, vitamins, minerals, and antioxidants, making them a valuable source of health-promoting components.	[225]
Peanut meal	Leftover after oil extraction from peanuts, peanut meal contains essential nutrients such as protein, fibre, and healthy fats, and bioactive compounds like polyphenols and antioxidants, which not only make it a valuable ingredient for poultry nutrition but also contribute to improved product quality, including enhanced meat flavour and nutritional value.	[226]
Almond shells	Almond shells are rich in essential nutrients and bioactive compounds, including fibre, antioxidants, vitamins, minerals, and polyphenols, making them a valuable and nutrient-dense agricultural by-product with potential applications in various industries including animal feeding, providing fibre.	[227]
Cottonseed meal	Cottonseed meal is rich in bioactive compounds such as gossypol and essential nutrients like protein, fibre, and minerals, making it highly suitable for poultry nutrition, resulting in improved product quality, enhanced growth, and optimal feed efficiency.	[228]
Rapeseed meal	Rapeseed meal is rich in essential nutrients such as protein, amino acids, and minerals, while also containing bioactive compounds like essential fatty acids, glucosinolates and phytosterols, making it highly suitable for poultry nutrition, promoting optimal growth, and enhancing the overall quality of poultry products.	[229]
Flaxseed meal	Flaxseed meal is a by-product rich in essential nutrients like protein, fibre, and healthy fats, as well as bioactive compounds such as lignans and alpha-linolenic acid, which enhance poultry nutrition and promote superior product quality, including enriched eggs with omega-3 fatty acids and improved meat lipid profiles.	[14,18]
Sesame meal	Sesame meal is a by-product from sesame oil extraction, and it is rich in essential nutrients such as protein, amino acids, vitamins E, minerals (calcium, phosphorus), along with bioactive compounds like lignans, phytosterols, and antioxidants, which contribute to its suitability for poultry nutrition, promoting enhanced product quality and overall health.	[223]
Pumpkin meal	Is a rich source of bioactive polyphenols, antioxidants, and essential fatty acids, which enhances egg quality and shelf-life.	[230]
Grains by-products		
Rice bran	Outer layer of rice, rich in fat, and used in swine and poultry feed. Rice bran is abundant in essential bioactive compounds and nutrients, such as antioxidants, tocopherols, tocotrienols, gamma-oryzanol, and various vitamins and minerals, making it a valuable and nutritionally rich ingredient with potential health benefits.	[231]
Wheat bran	Wheat bran is a valuable ingredient for poultry nutrition due to its rich content of bioactive compounds such as phenolic acids, flavonoids, and essential nutrients, including dietary fibre, vitamins, minerals, and proteins.	[231]
Brewer’s grains Distiller’s grains	Brewer’s grains and distiller’s grains are rich in essential nutrients, such as protein, fibre, vitamins, and minerals, while also containing bioactive compounds like antioxidants and beneficial enzymes, making them highly suitable for poultry nutrition.	[232]
Chickpea meal	Chickpea meal is rich in protein, fibre, vitamins, and minerals, and contains polyphenols and antioxidants, contributing to improved poultry nutrition and enhancing product quality with enhanced growth performance and health benefits.	[233]
Pea meal	Pea meal is rich in protein, fibre, vitamins, and minerals, and contains phenolic compounds and flavonoids, which contribute to its suitability for poultry nutrition and positively influence product quality, enhancing growth performance and promoting overall health.	[234]
Legumes by-products		
Potato peels	The outer skin of potatoes, containing carbohydrates and fibre, is rich in a diverse array of bioactive compounds and essential nutrients, including antioxidants, dietary fibre, vitamins (vitamin C and vitamin B6), minerals (potassium and magnesium), and resistant starch, making it a valuable source of nutritional benefits.	[235]
Sugar beet pulp	By-product from sugar production, containing fibre and carbohydrates. Sugar beet pulp contains a range of bioactive compounds, including polyphenols, flavonoids, betalains, and dietary fibres, which offer various health benefits to poultry, such as antioxidant properties, anti-inflammatory effects, and improved gut health.	[236]
Tomato pomace	The residual material after tomato juice extraction, which is rich in bioactive compounds, such as lycopene, phenolic compounds, vitamins, minerals, and dietary fibres, making it an excellent choice for poultry nutrition, enhancing product quality with improved antioxidant content and nutritional value.	[237,238]
Carrots waste	Carrot waste contains high amounts of residual bioactive, with currently little commercial value, but it provides carotenoids with important health implications.	[239,240]
Fruits by-products		
Apple pomace	Leftover after apple juice extraction, and is a source of fibre, vitamins, minerals, and antioxidants. Contains various polyphenols, including flavonoids such as quercetin, catechins, and epicatechins, as well as phenolic acids like chlorogenic acid and caffeic acid. These polyphenols contribute to the antioxidant properties and potential health benefits.	[241]
Citrus pulp and peel	Citrus peel is rich in essential nutrients and bioactive compounds, such as dietary fibre, antioxidants, polyphenols, and vitamins, making it highly suitable for poultry nutrition, promoting overall health, performance, and product quality.	[242,243]
Banana peels	The outer skin of bananas, which is rich in essential nutrients such as fibre, potassium, and vitamins, as well as bioactive compounds like polyphenols and antioxidants, making it a valuable and suitable component for poultry nutrition.	[244]
Pineapple pulp	The residue left after pineapple juice extraction, which contains bioactive compounds and essential nutrients such as bromelain enzymes, vitamin C, and essential minerals, rendering it highly suitable for poultry nutrition, promoting overall health and performance.	[245]
Watermelon rind	The outer green skin of watermelon is rich in essential nutrients such as fibre, vitamins, and minerals, along with bioactive compounds like citrulline and lycopene, making it highly suitable for poultry nutrition.	[246]
Mango peel	The outer skin of mangoes contains phenolics, carotenoids, and dietary fibre, vitamins, and minerals, making it a valuable and advantageous ingredient for poultry nutrition, contributing to improved immune function, antioxidant capacity, and overall performance in poultry.	[247]
Grape pomace	Contains bioactive compounds and essential nutrients, such as polyphenols, flavonoids, vitamins, and minerals, making it an ideal candidate for poultry nutrition, resulting in improved product quality with enhanced antioxidant properties and potential health benefits.	[248,249]
Rosehip meal	Rich in polyphenols, flavonoids, and carotenoids, along with essential nutrients like vitamin C, vitamin E, and dietary fibre, making it a valuable ingredient for poultry nutrition, leading to improved product quality, and enhanced antioxidant content, which enhances the product’s shelf-life.	[14,250]
Blueberry residue	Rich in anthocyanins, flavonoids, polyphenols, vitamins, and minerals, making it an excellent candidate for poultry nutrition, leading to improved product quality with enhanced antioxidant properties.	[251]
Cranberry waste	Contains flavonoids, phenolic acids, fibre, vitamins, and minerals, making it a highly suitable addition to poultry nutrition, leading to improved product quality and enhanced health benefits for the birds.	[9,252]

**Table 4:** European regulations on specific types of foods.

Type of Food	Regulation	References
food supplement	Directive 2002/46/EC states that food supplements are concentrated sources of nutrients or other substances with a nutritional or physiological effect, marketed in dose form (e.g., capsules, tablets, or sachets), and intended to supplement the normal diet. They correct nutritional deficiencies, maintain nutrient intake, or support physiological functions.	[279]
vitamins and minerals	Directive 2002/46/EC primarily focuses on vitamins and minerals, providing lists of harmonized vitamins, minerals, and their sources for food supplement manufacturing, along with labelling requirements. However, it does not fully cover other substances that may be present in food supplements. Additional compounds may be regulated by national rules or authorized under other EU legislations, such as regulations for the fortification of food, foods for specific groups, and novel foods.	[279]
enriched or fortified foods	Regulation (EC) No. 1925/2006 addresses enriched or fortified foods with specific nutrients. Unlike food supplements, these substances are added directly to food items to enhance their nutritional qualities. This regulation includes substances beyond vitamins and minerals, such as essential fatty acids, fibre, and various plants and herbal extracts.	[280]
foods for specific groups	Regulation (EU) 609/2013 refers to Foods for Specific Groups (FSG) and establishes requirements for various food categories, including infant formula, baby food, food for special medical purposes, and total diet replacement for weight control. It also sets rules for adding substances (including bioactive compounds) to these foods. The list of permissible substances includes minerals, vitamins, amino acids, carnitine, taurine, nucleotides, choline, and inositol.	[281]
novel foods	Regulation (EU) No. 2015/2283 on “novel foods” applies to foods not significantly consumed in the EU before 15 May 1997. This regulation covers new foods, food from new sources, new substances used in food, and new food production technologies.	[282]
food additives and nutrition and health claims	Regulations like (EC) 1333/2008 [28] for food additives and Regulation (EC) No. 1924/2006 for nutrition and health claims may affect foods containing additives or bioactive compounds with technological functions or those making specific nutritional or health claims, respectively. These regulations provide additional requirements and guidelines beyond those specifically related to food supplements and bioactive compounds.	[283,284]

## Data Availability

The data used to support the findings of this study can be made available by the corresponding author upon request.
